# Phylogeographic structure of the Common hamster (*Cricetus cricetus* L.): Late Pleistocene connections between Caucasus and Western European populations

**DOI:** 10.1371/journal.pone.0187527

**Published:** 2017-11-02

**Authors:** Natalia Yu. Feoktistova, Ilya G. Meschersky, Pavel L. Bogomolov, Alexandra S. Sayan, Natalia S. Poplavskaya, Alexey V. Surov

**Affiliations:** A.N. Severtsov Institute of Ecology and Evolution, Russian Academy of Sciences, Leninsky pr., Moscow, Russia; National Cheng Kung University, TAIWAN

## Abstract

The Common hamster (*Cricetus cricetus*) is one of the most endangered mammals in Western and Central Europe. Its genetic diversity in Russia and Kazakhstan was investigated for the first time. The analysis of sequences of an mtDNA control region and cytochrome *b* gene revealed at least three phylogenetic lineages. Most of the species range (approximately 3 million km^2^), including central Russia, Crimea, the Ural region, and northern Kazakhstan), is inhabited by a single, well-supported phylogroup, E0. Phylogroup E1, previously reported from southeastern Poland and western Ukraine, was first described from Russia (Bryansk Province). E0 and E1 are sister lineages but both are monophyletic and separated by considerable genetic distance. Hamsters inhabiting Ciscaucasia represent a separate, distant phylogenetic lineage, named “Caucasus”. It is sister to the North phylogroup from Western Europe and the contemporary phylogeography for this species is discussed considering new data. These data enabled us to develop a new hypothesis to propose that in the Late Pleistocene, the continuous range of the Common hamster in the northern Mediterranean extended from the central and southern parts of modern France to the Caucasus; however, its distribution was subsequently interrupted, likely because of climate change.

## Introduction

One of the topical problems of ecology in the 21th century is the global decline of biodiversity [1]. The major challenge is to better understand the responses of ecosystems to realistic scenarios of biodiversity change caused by the simultaneous processes of extinction [2] and invasion. It is believed that species with small ranges and/or low abundance are most likely to be endangered [3]. Such species are most sensitive to global climate changes and anthropogenic pressure, and are more susceptible to epizootics [4]. Wide-ranging and previously abundant species are thought to be more robust to the negative consequences of these effects, however a few species have suffered catastrophic collapse despite their large range and/or abundance. One well-known example is the passenger pigeon (*Ectopistes migratorius*), once possibly the most numerous bird species in the world, completely vanished by the beginning of the 20th century. A similar event occurred in two other previously abundant birds species, the yellow-breasted bunting (*Emberiza aureola*) and the rustic bunting (*E*. *rustica*) [5, 6]. Among mammals, the most dramatic case is that of the Common hamster (*Cricetus cricetus* Linnaeus, 1758). This species had one of the largest ranges of all the Palearctic mammal fauna, extending nearly 6 million km^2^, from Belgium to the Krasnoyarsk Province (Russia). The Common hamster inhabits forest-steppes and steppes, but is strongly attracted to anthropogenically modified habitats such as agriculture areas, vegetable gardens, and farm enterprises. Over the last 70 years, the abundance of the Common hamster has been significantly reduced. As an agricultural pest and commercial species, it has been harvested by the millions in the 1950s and 1960s [7] and has become one of the most endangered mammalian species in Europe [8]. This reduction is most pronounced in Western and Central Europe, where its previous range decreased by 75% due to intensive fragmentation [8–18]. The species was included in Appendix II (protected species) of the 1979 Bern Convention on the Conservation of European Wildlife and Natural Habitats, and since 1992, it has been listed in Appendix IV of the Habitats Directive, which provides strict legal protection in all EU countries [14]. The causes of the global decline of the Common hamster are complex and not yet fully understood.

Captive breeding is essential for reestablishment of populations of Common hamsters in regions where it has vanished from natural habitats. However, not all regions with high population densities of the hamster are appropriate as donor regions. Although caged breeding and reintroduction actions are guided by genetic aspects to some extent, many questions concerning the genetics of hamster reintroductions in some countries remain [19]. Genetic diversity *per se* is a very important index of population health and stability. Therefore, molecular genetic studies and phylogeographic analysis of the hamster populations are not only of theoretical interest but also necessary for species restoration efforts. Studies over the last 10–15 years are numerous but apply only to the western region of the Common hamster range. In 2003, the data regarding allelic variation in the major histocompatibility complex (MHC) gene in hamsters belonging to almost extinct populations from France and the Netherlands was documented [20]. This study, coupled with the results of other research that evaluated allelic diversity at microsatellite loci [19–22], revealed substantial declines in genetic variation in populations from Western and Central Europe. Based on mtDNA sequencing the presence of two phylogenetic lineages, henceforth "Pannonia" and "North", of Common hamsters inhabiting Western and Central Europe was discovered [11]. The time of divergence of the two lineages was estimated as 85–147 kya. The core area of the range of the phylogroup Pannonia is the Pannonian Plain surrounded by the Carpathians, Alps, Dinaric Alps, and Balkan Mountains. The hamsters of the phylogroup North inhabit Western Europe and represent two lineages, the Western (Belgium, France, the Netherlands, and southern Germany) and Central (northern regions of Germany). A recent study [23] also detected the presence of a haplotype of the Central phylogroup in southwestern Poland. The time of isolation of these groups was estimated as 10–15 kya, at the end of the Last Glaciation Maximum. Mitochondrial genotypes of hamsters from Russia and Poland could not be attributed to any of the aforementioned groups, and did not represent a distinct group. Some later members of the E1 group, distanced from both the Pannonia and North mitochondrial phylogroups, were revealed in Poland [24, 25]. Mitochondrial haplotypes of several animals from Russia, obtained earlier, were close to this group, but the sequences did not form a single clade. In the southeastern Poland portion of the species range, haplotypes of Pannonia and E1 lineages inhabit areas located very close to each other [26, 27]. Authors of these studies cited above and some later publications agreed with K. Neumann and suggested that the genetic differentiation among European hamsters was mainly caused by immigration from different eastern refuges and was a more recent event. Possible source populations are likely in the Ukrainian and the southern Russian plains, core areas of hamster distribution [11]. Ancestors of the phylogroup North, distributed now over Western Europe, possibly also expanded from the east. However, source location for the immigration and its route are not clear, considering the absence of the North phylogroup haplotypes in Poland. The phylogenetic group E1 has been reported only from Poland and the Ukraine, and the Ukrainian steppe is likely its refugium [28]. Thus, despite the considerable number of studies, the history of the formation of the phylogeographic structure of the Common hamster in Central and Western Europe remains unclear. This is because of a conspicuous lack of data, not only from the steppe regions of Ukraine and southern Russia (considered as potential refugia), but also from the entire eastern portion of the species range. This portion of the species range, as mentioned above, is approximately three times larger than that of Central and Western Europe. In addition, there are numerous areas in Russia where many European mammalian species survived the unfavorable climatic periods of the Pleistocene. These include regions such as the Caucasus, Urals, Upper Dnieper, and Central Russian Upland [29–33]. Until now, these areas, primarily the Caucasian and Urals, have been sustainable centers of mammalian biodiversity [29].

The goal of the present study was to clarify the relationships between the phylogenetic lineages known for well-studied western and nearly unexplored eastern part of the species range and evaluate the phylogeographic structure and genetic diversity of the Common hamster in Russia and Kazakhstan.

## Materials and methods

**Fig 1:**
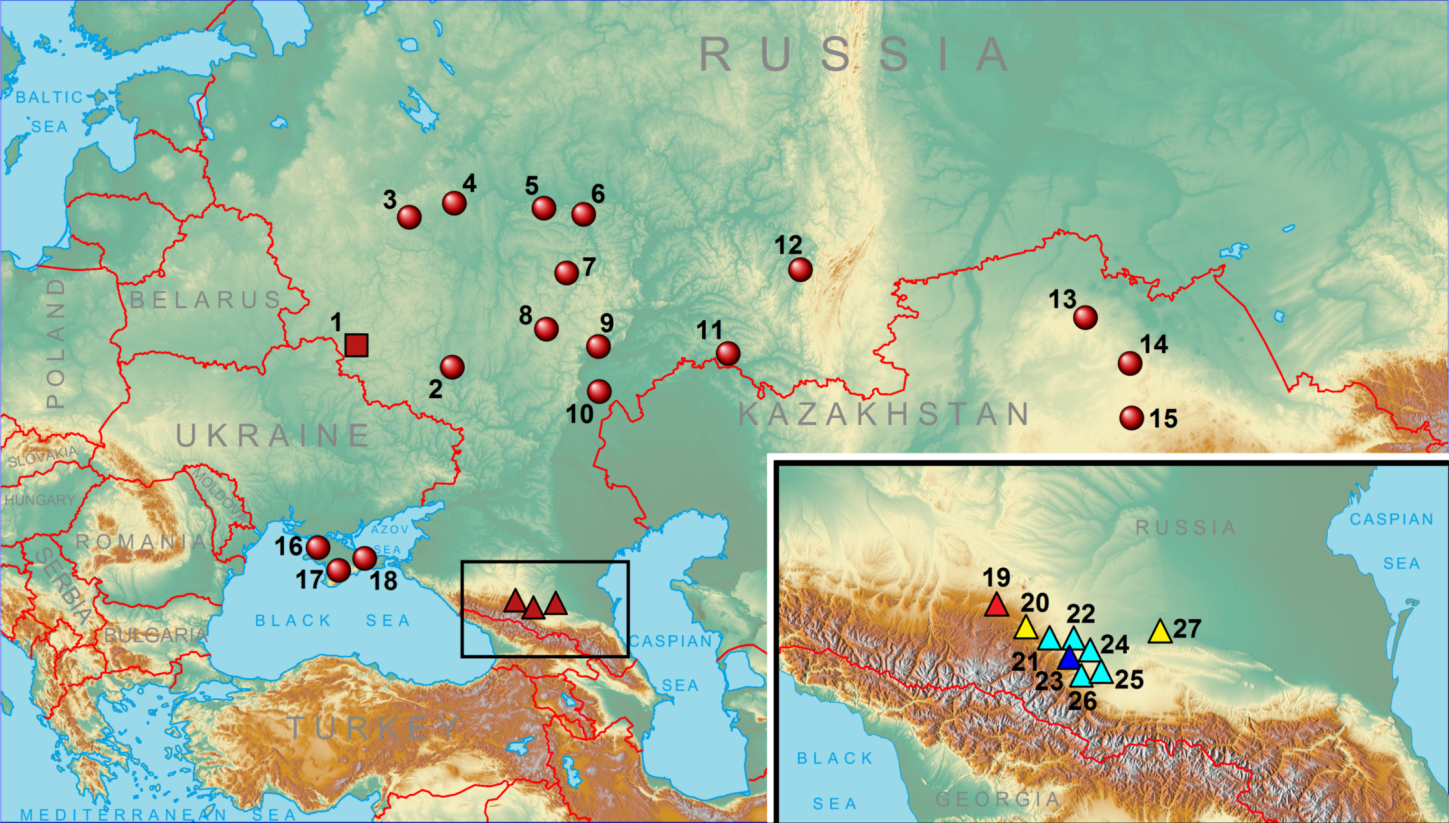
Sample distribution map. Bryansk Province in Western Russia is indicated by a square, localities from the Ciscaucasian area by triangles, and the others by circles. In the breakout: Nalchik City is shown by dark blue, the Nalchik surrounds (restricted by 25-km radius) by light blue, Kislovodsk by red, and other localities by yellow.

We analyzed fragments of the mitochondrial genome of 60 Common hamsters originating from the Ciscaucasian region (Russia): 26 specimens from Nalchik (Kabardino-Balkariya Republic, KBR) and the surroundings in a radius of 25 km, 33 specimens from Kislovodsk, Stavropol Province), Russia and the closest surroundings, and one specimen from Kamennomostskoye Village (Zolsky District of KBR). We also analyzed 12 specimens from central Russia (namely, four hamsters from Moscow, three from the area surrounding Suzdal City, four from the area surrounding Nizhny Novgorod City, and one from the Republic of Mordovia), one specimen from Ural region (Orenburg Province), three from northern Kazakhstan and eight specimens from different districts of Crimea (Table 1, Fig 1).

**Table 1:** List of samples localities. Point numbers correspond to Fig 1.

Point number	Geographical location	Coordinates
1	Western Russia, Bryansk prov., Novoyamskoye village	52°11'N, 34°32'E
2	Central Russia, Voronezh prov., Khleborodnoye village	55°38′N, 37°43′E
3	Central Russia, Moscow City	55°38′N, 37°43′E
4	Central Russia, Vladimir prov., Pavlovskoye village	56°19′N, 40°28′E
5	Central Russia, Nizhny Novgorod prov., Afonino village	56°16′N, 44°6′E
6	Central Russia, Nizhny Novgorod prov., Kekino village	55°54′N, 46°1′E
7	Central Russia, Mordovia Republic, Ozyorny village	54°14′N, 45°17′E
8	Central Russia, Penza prov., Privolzhskaya Lesostep Nature Reserve	52°50'N, 44°27'E
9	Central Russia, Saratov prov., Slavyanka village	51°50′N, 46°15′E
10	Central Russia, Saratov prov., Dyakovka village	50°43′N, 46°47′E
11	Ural, Orenburg prov., Tashla village	51°46′N, 52°44′E
12	Ural, Bashkortostan Republic, Krasnousolsky village	53°54'N, 56°28'E
13	Nothern Kazakhstan, Akmola prov., Shchuchinsk city	52°55'N, 70°16'E
14	Nothern Kazakhstan, Akmola prov., Turgay village	51°46′N, 72°44′E
15	Nothern Kazakhstan, Karaganda prov., Temirtau city	50°8′N, 72°52′E
16	Crimea, Rozdolne	45°46′N, 33°30′E
17	Crimea, Simferopol city and vicinities	44°58′N, 34°6′E
18	Crimea, Yerofeyeve village	45°12′N, 35°39′E
19	Caucasus, Stavropol prov., Kislovodsk city and vicinities	43°55′N, 42°43′E
20	Caucasus, Kabardino-Balkar Republic, Kamennomostskoye village	43°44′N, 43°3′E
21	Caucasus, Kabardino-Balkar Republic, Islamei village	43°40′N, 43°27′E
22	Caucasus, Kabardino-Balkar Republic, Dygulybgei village	43°40′N, 43°32′E
23	Caucasus, Kabardino-Balkar Republic, Nalchik city and vicinities	43°30′N, 43°39′E
24	Caucasus, Kabardino-Balkar Republic, Stary Cherek village	43°28′N, 43°51′E
25	Caucasus, Kabardino-Balkar Republic, Argudan village	43°25′N, 43°55′E
26	Caucasus, Kabardino-Balkar Republic, Zaragizh village	43°20′N, 43°43′E
27	Caucasus, North Ossetia-Alania Republic, Mozdok city	43°45′N, 44°40′E

DNA was extracted from preserved tissues in 96% alcohol that were collected from fresh, naturally deceased animals. For total DNA extraction, we used KingFisher™ Flex Magnetic Particle Processor (Thermo Scientific) and InviMag Tissue DNA Mini Kit/KF96 (STRATEC Molecular) kit or (for singles samples) reagent kit Diatom DNA Prep 100 (Isogene Lab. Ltd., Moscow, Russia) according to the manufacturers' instructions. The resulting DNA solutions were stored at -18°C.

Subsequent PCRs were conducted using the master mix MagMix 2025 (Dialat Ltd., Moscow, Russia). Primers were synthesized at JSC Syntol (Moscow). For markers of phylogenetic lineages, we used sequences of a mitochondrial control region and the cytochrome *b* (*cytb*) gene. For amplification of the control region, we used primers H00651 and DL2, described earlier [34] and for *cytb*, we used the primers Cricetus_cytbF: 5′-AACCATGCGTTCATTGATCT-3′ and Cricetus_cytbR: 5′-CAATTATGCTAGCGATTGGTATAAA-3′.

The purified PCR products were used as a template for sequencing reactions using the BigDye Terminator v. 3.1 kit (Applied Biosystems, US) according to the manufacturer’s instructions. Sequencing was conducted on a Genetic Analyzer 3500 (Applied Biosystems, US). Each sample and DNA locus was sequenced twice: setting the reaction with forward and reverse primers. Quality control of the automatic decoding of chromatograms, the merging of forward and reverse individual sequences, and their alignment and storage was completed using BioEdit v.7.2.5 software [35].

For all the above samples, we obtained *cytb* sequences that were 924 bp in length, corresponding to positions 97–1020 of the complete gene sequence, and control region sequences 879 bp in length, starting from the first position (alignment performed for the mitochondrial genome of *Mesocricetus auratus*, GenBank EU660218). The total length of the concatenated fragments was 1803 bp. For data analysis, we used previously obtained sequences for six hamsters from the Ciscaucasian region (three specimens from Nalchik and surroundings, one specimen collected in the area surrounding Mozdok (Republic of North Ossetia) and two specimens from Stavropol Province (without detailed locality information, these samples are not shown in Fig 1), as well as data from 42 hamsters from Crimea, nine specimens from different regions in central Russia, one specimen from Ural region, and nine specimens from northern Kazakhstan [34] (Fig 1, Table 1).

Unique sequences (haplotypes) of the cytochrome *b* gene and the control region obtained during this study were deposited in GenBank under accession numbers KY748063–KY748092 and KY795998. The resulted unique combinations (1803 bp) of haplotypes of *cytb* and control region are shown in Table 2.

**Table 2:** Accordance of designation of haplotypes of concatenated mtDNA fragment (1803 bp) shown on Fig 2 and *cytb* gene and control region sequences deposited in Genbank.

Haplotype designation	GenBank Ac.No. (*cytb*+dloop)
08Stav	KF271753 + KF271771
43Mozd	KF271755 + KF271770
99Stav	KF271754 + KF271771
003Nal	KY748069 + KY748087
887Arg	KR706038 + KY748085
886Arg	KR706038 + KY748086
016Nal	KR706038 + KR706044
869Kis	KY748067 + KY748085
002Nal	KR706037 + KR706043
894Nal	KY748062 + KY748080
401Nal	KY748062 + KY748083
405Nal	KY748062 + KY748081
006Nal	KY748071 + KY748081
848Nal	KY748063 + KY748081
402Nal	KY748064 + KY748083
004Nal	KY748070 + KY748083
400Nal	KY748066 + KY748081
898Nal	KY748064 + KY748082
005Nal	KR706038 + KR706043
874Kis	KY748066 + KY748083
852Kis	KY748065 + KY748084
007Nal	KY748072 + KY748088
48Mosc	KF271752 + KF271766
32Mosc	KF271752 + KF271767
002SmP	KR706041 + KF271780
101ZuC	KR706041 + KF271779
050RzC	KF271756 + KF271776
402RzC	KF271756 + KF271778
040SmS	KF271756 + KF271779
401RzC	KF271756 + KF271777
200Ker	KY748079 + KY748090
013Sar	KF271757 + KF271764
104PrL	KR706035 + KR706042
105PrL	KR706036 + KR706042
57Turg	KF271760 + KF271774
12Turg	KF271762 + KF271775
38Novg	KF271763 + KF271765
16Turg	KF271761 + KF271773
948Ore	KY748077 + KY748093
250Url	KR706039 + KR706045
998Shc	KY748075 + KY748091
997Tem	KY748076 + KY748092
996Tem	KY748076 + KY795998
165Sar	KF271758 + KF271772
05Mord	KY748073 + KY748089
03Vorn	KR706040 + KF271769
10Brnk	KF271759 + KF271768

The phylogenetic analysis of sequences from the western and eastern parts of the Common hamster range was conducted based on the *cytb* gene sequence of 904 bp length, as it was the most numerous in GenBank.

In addition to sequences noted above we used records AJ633756–AJ633782 [11]; AJ973392 [12]; EU107523–EU107535 [25]; KF271752–KF271761 [36]; KR010651–KR010664 [37]; KT224635–KT224640 [38], 91 sequences in total. For the phylogenetic analysis, we used the Bayesian algorithm using MrBayes 3.2 [39, 40] for 5,000,000 iterations and 100,000 iterations of burn in. For the analysis of concatenated *cytb* and control region sequences, the homologous sequences of *Allocricetulus eversmanni* (GenBank KP231506) were used and for 904 bp *cytb* fragments only, we used homologous sequences of *A*. *eversmanni* (KP231506) and *Cricetulus migratorius* (AJ973387) as outgroups. In both cases, as well as for divergence time estimations, HKY + I + G model parameters were used. This model was chosen based on the analysis using the MEGA7.0.20 program [41, 42] using the Bayesian information and corrected Akaike information criteria. The corrected Akaike information criterion values were also calculated using MrModeltest 2.3 software [43].

We also used MEGA software to calculate within- and between-group averaged genetic distances and determined differences and the diversity of groups forming well-supported clades in a phylogenetic tree, as well as of groups representing separate geographic regions. For both evaluations, the phylogenetic aspect was key. Thus, the analysis was completed for sets of unique sequences (haplotypes), irrelative of their frequency in population samples. For the construction of a haplotype network using the Median Joining algorithm we used Network 5.0.0.0 (www.fluxus-engineering.com) [44].

Divergence times were evaluated in BEAST v2.4.5 [45] based on 904 bp *cytb* fragment accepted as a single partition. We added sequence of *Tscherskia triton* (AJ973388) to our *cytb* alignment and used divergence time evaluated for *Tscherskia triton* and *Cricetulus migratorius*, *Allocricetulus eversmanni* and *Cricetus cricetus* clade [12] as a calibration point. The values given in referred article (mean 6.7 mya, SD 1.4 mya) were used as parameters of a normal probability distribution. As our phylogenetic analysis showed that all *cytb* haplotypes known today for Pannonia and all other *Cricetus cricetus* haplotypes represent two well-supported monophyletic clades, we assumed that the time estimated earlier [11] for separation of Pannonia (split between Pannonia and North clades) may be applicable for the two extended clades as well. So, we used the proposed averaged range, 85–147 kya, as a calibration point for divergence of Pannonia clade and all other haplotypes as bounds of a uniform probability distribution. The analysis was run with relaxed clock and a Yule speciation process. Chain lengths were 50 million, sampling trees and parameter estimators every 1000 generations with the first 20% discarded as burn-in. Log files were checked for effective sample sizes (ESS) of more than 200 and convergence of posterior values in Tracer v1.6.0 (http://tree.bio.ed.ac.uk/software/tracer/). Trees were summarized as maximum clade credibility trees using TreeAnnotator, version 2.4.5 [45] and visualized using FigTree, version 1.4.3 (http://tree.bio.ed.ac.uk/software/figtree/).

Field collection in Russia and Kazakhstan was carried out under the fulfillment of the governmental program "Animal communication–behavioral and physiological adaptations” (№ 0109-2014-0017), with governmental permission to collect such samples from public property. Since all the samples were collected exclusively from recently deceased animals that were found dead, ethics committee approval was not needed.

## Results

**Fig 2:**
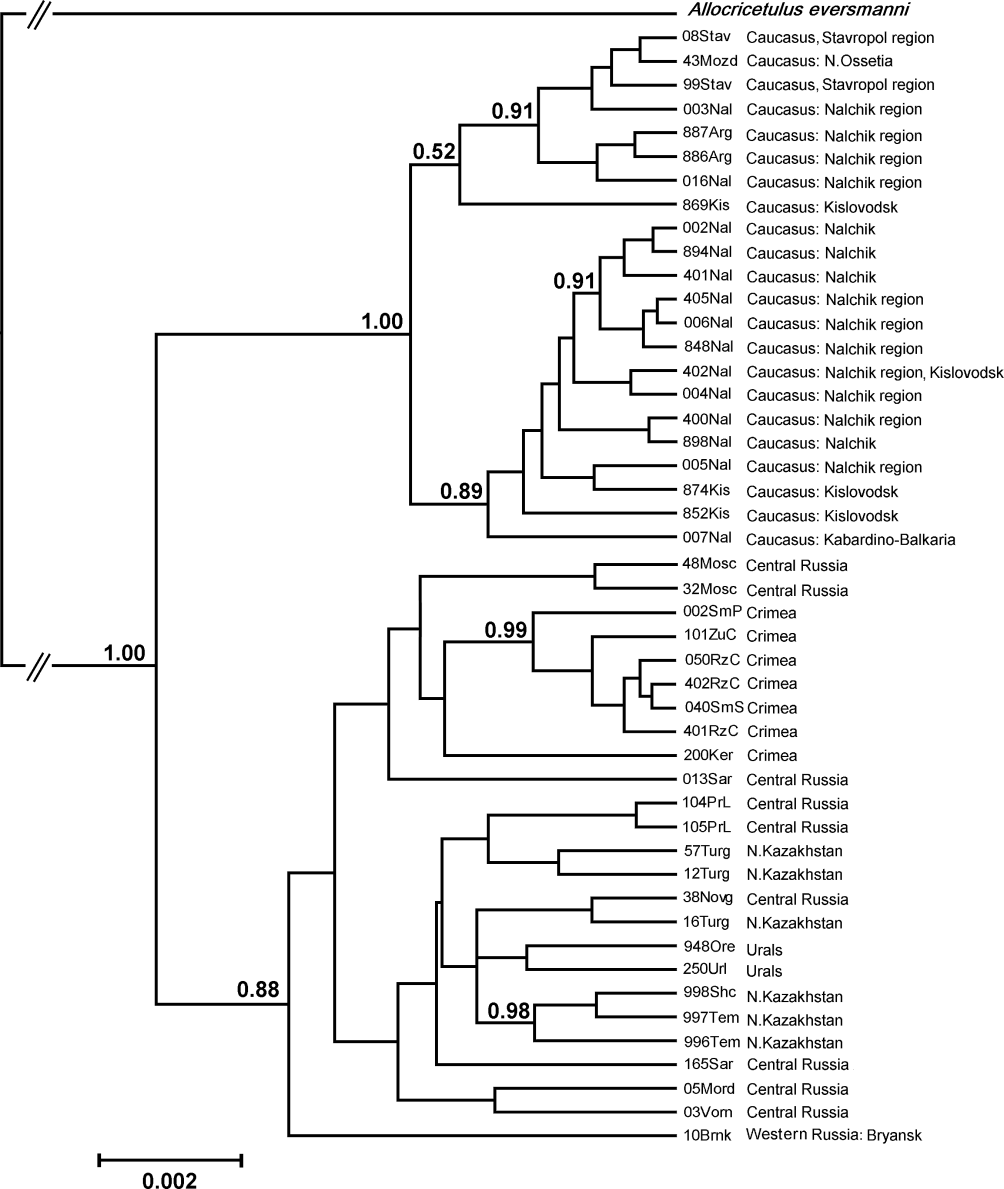
Cladogram resulting from Bayesian phylogenetic analysis of haplotypes of concatenated sequences of *cytb* gene and control region for hamsters within the investigated area. Support values are given if they exceeded 0.5 for nodes that included three or more haplotypes. For GenBank accession numbers see Table 2.

The phylogenetic analysis of the two mtDNA fragment concatenated sequences (1803 bp) revealed two distanced clades present within the investigated area. Sequences found in the Ciscaucasian region (22 haplotypes for 66 analyzed specimens) formed a separate well-supported haplogroup and sequences known for territories of central Russia (10 haplotypes for 17 hamsters), the Ural region (two haplotypes for two specimens), Crimea (seven haplotypes for 50 specimens), and northern Kazakhstan (six haplotypes for six specimens); totaling 25 haplotypes for 75 analyzed specimens) formed the other clade (Fig 2).

The mean of genetic distance evaluated for the haplotype sets corresponding to the two clades was estimated as 1.005%. The number of polymorphic sites within 1803 bp sequences was 29 for the Ciscaucasian region (transitions/transversions ratio was 2.63) and 57 (transitions/transversions ratio 3.54) for sequences for hamsters originating from other investigated regions. No indels were found in the control region sequences.

The Ciscaucasian lineage was notably diverse: among 47 haplotypes described for the total investigated area, 22 were found in the Ciscaucasian region. The mean of within group genetic distance evaluated for the Ciscaucasian lineage was estimated as 0.340%, whereas the same index evaluated for the other phylogroup was 0.521%, despite the much larger distribution area.

**Fig 3:**
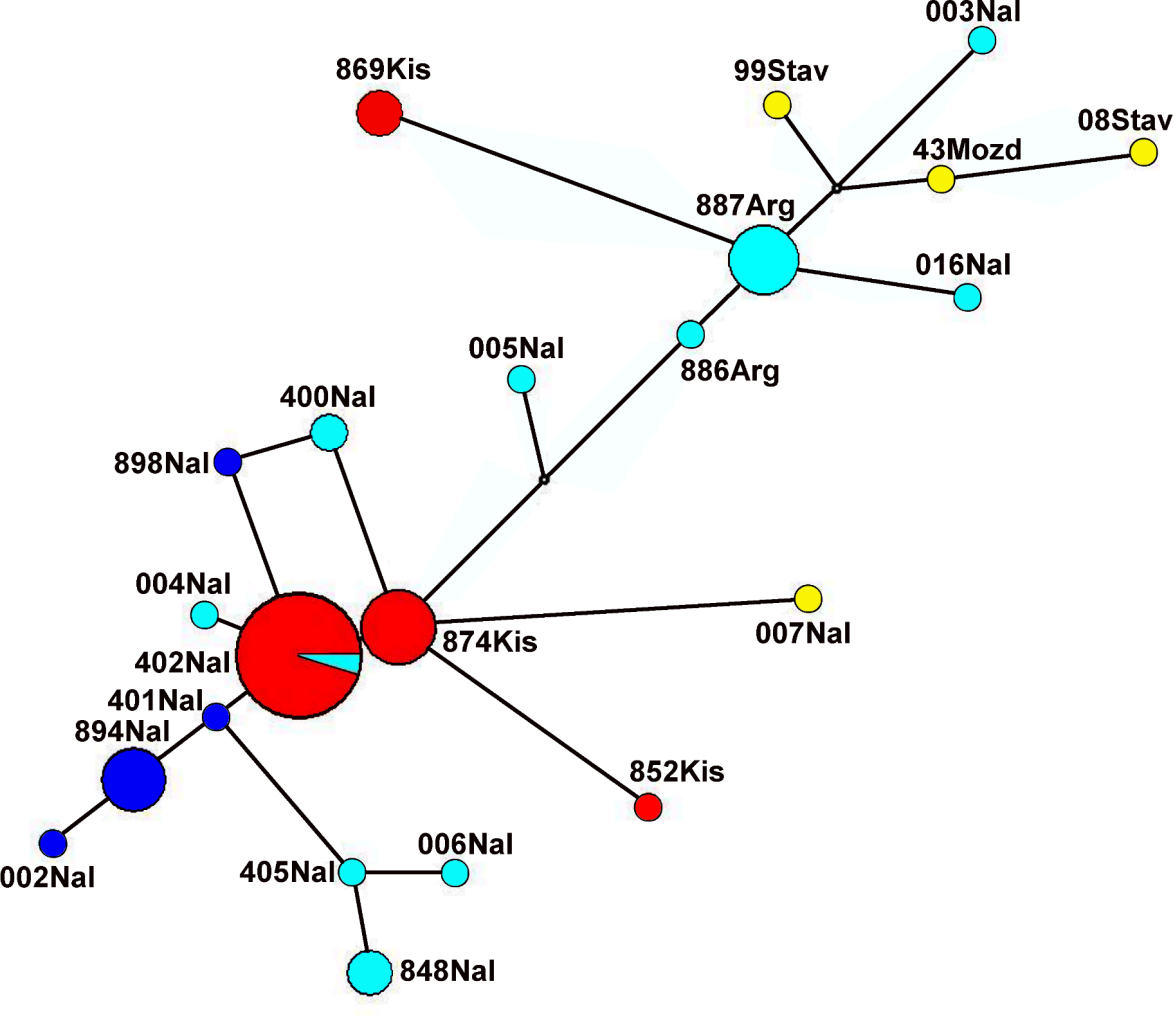
Median-joining Network of concatenated of cyt*b* gene and control region sequences found in the Ciscaucasian region. Haplotypes found in Nalchik City are marked in dark blue, in the Nalchik City surrounds (restricted to a 25-km radius) by light blue, in Kislovodsk City by red, and in other localities by yellow. Minimal distances between circles correspond to one nucleotide substitution and circle diameter with the haplotype frequency.

At the same time, no geographic substructure was found within the Ciscaucasian lineage; haplotypes clustered together on the phylogenetic tree or on the median network occurred in different localities (Fig 3).

In addition, no geographic substructure were found for the second lineage that consisted of haplotypes found in central Russia, the Ural region, northern Kazakhstan, and Crimea. Closely related sequences were found in distant regions. Originality was found for some haplotypes in Crimea: six of them formed a well supported (PP = 0.99, Fig 2) clade of a low within group distance (0.141%). However, together with another Crimean haplotype (200Ker), the mean of within group distance increased to 0.201% and the clade uniting all seven haplotypes for Crimea was not supported.

**Fig 4:**
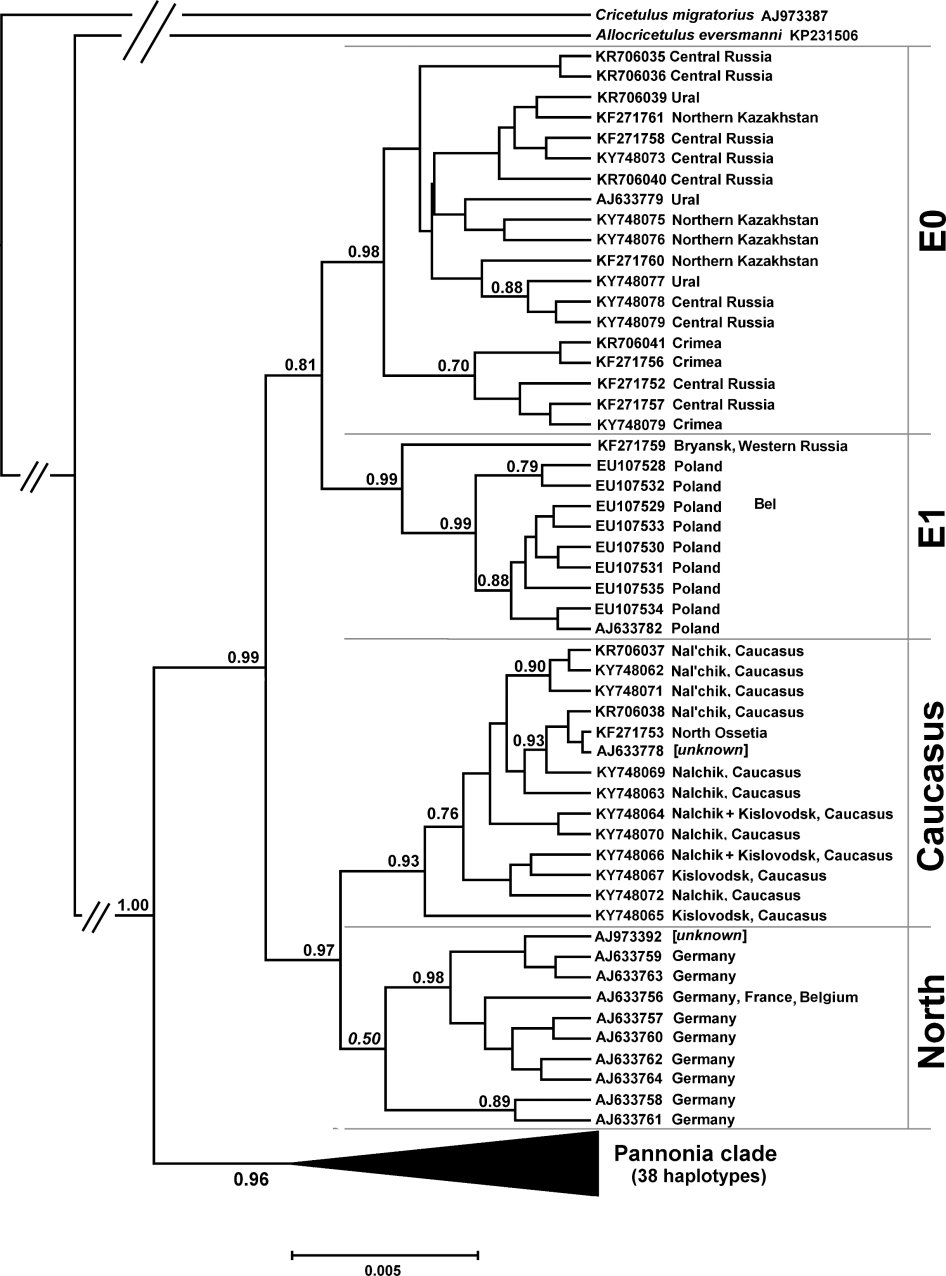
Phylogram resulting from Bayesian phylogenetic analysis of 904 bp cyt*b* gene fragment haplotypes. Support values are given if they exceeded 0.5 for nodes that included three or more haplotypes.

To evaluate the relationships between mtDNA lineages known for western and eastern parts of the Common hamster range, we conducted a phylogenetic analysis based on sequences of a 904-bp fragment of the cytochrome *b* gene. The results confirmed that haplotypes known for all specimens collected in territories from the Moscow region in the west to Akmola and Karaganda provinces of Kazakhstan in the east formed a single monophyletic clade, with the mean interhaplotypic distance of 0.444% (Fig 4).

This phylogroup, denoted as E0, seems to be a sister clade for the group of haplotypes reported from Poland and named earlier the E1 lineage [25, 26]. The haplotype of hamsters originating from Bryansk Province in Western Russia belonged to E1, not the E0 clade. The mean interhaplotypic distance in E1 clade was 0.334% and mean of intergroup difference for E0 and E1 haplotypic sets was 0.986%.

The existence of separate monophyletic clade formed by haplotypes found for Common hamsters inhabiting Ciscaucasius was also confirmed by extended sequence set analysis. The mean of interhaplotypic distance within this lineage, named "Caucasus" was 0.345%. According to the analysis results, the Caucasus lineage is a sister to the clades uniting haplotypes from Western Europe (Germany, France, and Belgium) which are known as the North phylogroup [11]. This group is evidently heterogeneous and, in our case, was not supported as a single clade. Nevertheless, as the unity of all *Cricetus cricetu*s lineages known for Western Europe as a group separated from Pannonia and E-type sequences was confirmed earlier [11, 25], we keep "North phylogroup" or "North-type lineages" definition in our discussion below.

The mean of between group distance for the Caucasus lineage and the clade uniting all North-type haplotypes was 0.917%. This value is comparable to the distance found for E0 and E1 lineages– 0.986%. Caucasus and all North-type haplotypes together also form a monophyletic clade, which is a sister for a clade uniting all E-type haplotypes. The mean interhaplotypic distances within the two "large" clades were also similar: 0.686% for E group (as E0 + E1) and 0.647% for the "Caucasus + North" clade. The mean intergroup distance for E and "Caucasus + North" clades was 1.118%.

Finally, the two large clades were found to be sisters and may be united into a single well-supported group of the next order, which in turn, is separated from all haplotypes of the Pannonia lineage by 1.524%. It is notable that the two super clades had a comparable intragroup diversity level in our analysis; averaged interhaplotypic distances were 0.801% for Pannonia-type haplotypes and 0.896% for haplotypes outside the Pannonia group range.

## Discussion

This is the first evidence to demonstrate the population of the Common hamster occurring in the study area of Russia and northern Kazakhstan is represented by at least three distanced and well-supported phylogenetic lineages. The Ciscaucasian region is inhabited by populations belonging to a lineage named "Caucasus". Hamsters of the E1 lineage, previously reported from some areas of the Ukraine and Poland, were found in the Bryansk Province (Western Russia). The E0 lineage is widely distributed, from Crimea through the Eastern European Plain and Ural region to northern Kazakhstan.

The fact that Common hamsters populated the Ciscaucasian region belonged to a separate group is not surprising. Satunin (1909) described a specimen of the Common hamster from Caucasus and separated it into a subspecies, *Cricetus cricetus stavropolicus* [46]. Later Ognev (1924) [47], analyzed numerous specimens, confirmed its subspecies status. He noted that the skull morphology and body dimensions, as well as the pelage color of *C*. *c*. *stavropolicus* differed in particular from *С*. *с*. *tauricus* Ognev, 1924 that occurred in the territory between the Don and Dnieper Rivers and in Crimea.

It is noteworthy then northern Caucasus is known to be a refuge region characterized by a high level of biodiversity and the presence of numerous endemic species [29]. The Common hamster is widely distributed there, inhabiting foothills, steppes, stepped habitats along mountainsides, and agricultural landscapes. In addition, the hamsters actively populate urban and rural settlements. According to the analysis of the collections of data from the Institute of Ecology of Mountainous Territories in Nalchik, the Common hamster occurs to a height of 1150 m a.s.l. Specimens used in the present study were captured at 380–950 m a.s.l.

The phylogenetic relationship between the mitochondrial lineages, which occur in Ciscaucasius and Western Europe, are the basis for a reexamination of the recent history of the species range. It is accepted that during the Late Pleistocene, the Common hamster extended its range following the formation of the open steppe habitats that are typical of moderate glacial intervals and cooler phases during interglacials [11]. Correspondingly, during warmer phases interglacial periods and extension of forest habitats, the hamster range had been split into two separated refuge areas.

The separation of the Common hamster population which might have been caused by landscape dynamics took place in the Pannonia Basin presumably 147–85 kya [11], during the period corresponding to the last (Riss-Würm) interglacial or the MIS-5 epoch. Later, the Pannonian Basin habitat remained suitable for the hamsters during the Late Pleistocene climate changes. As a result, the most distanced (known) phylogroups of Common hamsters were formed. To date, this phylogroup has been endemic, although its range enlarged and now covers a broad area.

More northern regions of modern Poland and countries of Western Europe were presumably inhabited by hamsters that expanded from eastern refuge areas. However, during later periods of global warming, the species abandoned a large part of this territory [11, 25–28]. It may be suggested that the ancestor of the phylogenetic lineage, which later formed both the recent Caucasus and North groups, immigrated into the European territories in the same way, i.e., expanding from the East European Plain. This suggestion, however, is in contradiction with the absence of North-type haplotypes within both the E1 and Pannonia group recent ranges. As well, nobody found hamsters of the North phylogroup in Ukraine, and central Russia. The recent finding of the North-type haplotype in Poland [23] represents an isolated population in the southwestern region of Poland, 300 km from eastern Germany, which is populated by North phylogroup hamsters. The authors suggested that this occurred because of recent migration or fragmentation of the North phylogroup's range, which earlier included southwestern districts of Poland, but continued to be allopatric with the E1 range located distinctly eastwards. Thus, this discovery does not affect the general pattern of European Common hamster phylogeography.

Dating provided by K. Neumann *et al*. [11] refers to the most recent common ancestor for the Pannonia phylogroup and all other known lineages to the period of 147–85 kya. This period correlates with the last (Riss-Würm) interglacial or the MIS-5 epoch, i.e., climatic conditions that presumably lead to isolation of groups of hamster populations in separate refuge areas. According to our analysis, the genetic distances between E0 and E1 as well as between Caucasus and North phylogroups are approximately 57–67% of the distance between each of these four clades and the Pannonia lineage. Thus, the divergence of haplogroups recently occurred in territories outside the Pannonia phylogroup range should be dated later than the MIS-5 epoch. The MIS-3 epoch, which began approximately 57 kya, was associated with interstadial warming, and appears to be appropriate for this event(s).

The accurate estimations of divergence times essentially depend on chosen calibration points, models and genetic markers used, and are the subject of a separate study (Meschersky *et al*., in prep.). Nevertheless, the results obtained using one of the possible approaches do not contradict our suggestion (Table 3).

**Table 3:** Divergence time (kya) between the Common hamsters phylogenetic lineages as evaluated based on suggested (1) *Tscherskia triton and (Cricetulus migratorius + Allocricetulus eversmanni + Cricetus cricetus)* clade and (2) Pannonia and North-type lineages separation time.

Node	Median	95% HPD
E0+E1	67	37–104
E0	51	25–84
E1	48	20–85
Caucasus+North	63	32–98
Caucasus	43	20–74
North	49	21–83

The present *Cricetus cricetus* range located to the west from Volga River is bounded on the south by the Greater Caucasus Mountain Range, the northern coast of the Black Sea, Balcan Mountains, and Dinaric Alps. However, during the Late Pleistocene, Common hamster remains have been reported from more southern areas adjoining the Mediterranean. In the Lazaret cave in southeastern France, the remains of this species were found at levels correlated with times earlier than the MIS-5 epoch and later [48]. During the end of the Riss-Würm interglacial and beginning of Last Glacial Maximum (LGM), i.e., MIS-4 –MIS-3 epochs, approximately 70–25 kya, remains of the Common hamster have been reported from several locations in the Apennine Peninsula [49]. In addition, Late Pleistocene (Vistulian) remains of Common hamsters have been reported from Croatia, France, Italy, Slovenia [50], and Serbia [51].

According to modern paleogeography data obtained using a complex of dating methods, during the MIS-5 epoch, the interglacial period, and the transition to the MIS-4 glacial, the Bosporus strait was open, the Black Sea basin was vast, and the Crimean Peninsula was separated from the mainland by the Sea of Azov, which in turn, was connected with the Caspian Sea through the Manych Strait. The Manych Strait also disjointed the Caucasus from East European Plain. However, later, during the first stage of the MIS-4 epoch, followed by extensive glaciation (about 70–57 kya), the Black Sea basin regressed (Post-Karangat regression) and the Bosporus strait disappeared. In addition, the Manych Strait ceased to exist and Crimean Peninsula was connected to the Caucasian region by dry land. This orographical state lasted during the interstadial warming (MIS-3 epoch, approximately 57–25 kya) and the beginning of the LGM or MIS-2 epoch [52].

**Fig 5:**
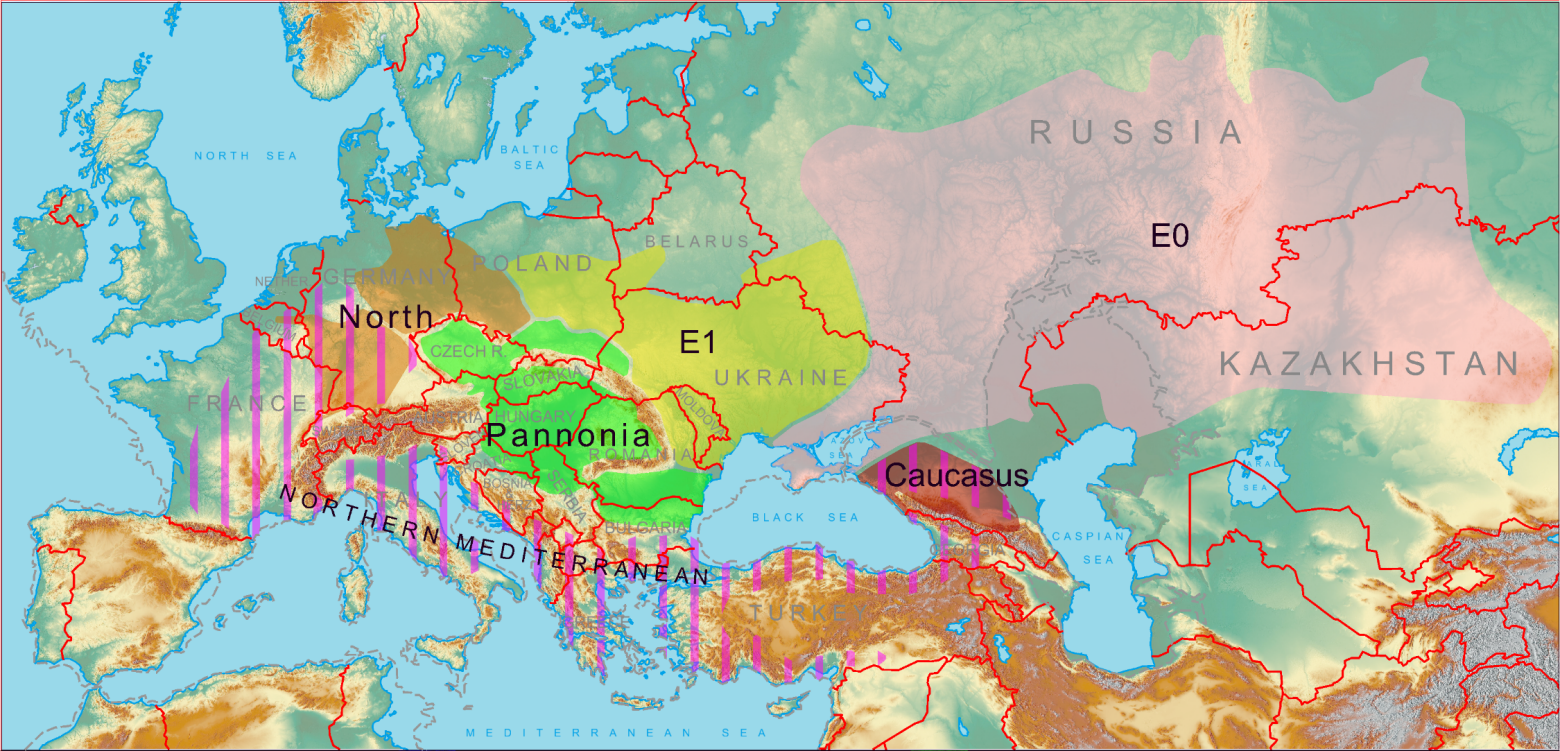
The hypothetical ranges of the Common hamster hyplogroups: Vertical hatching–Late Pleistocene Northern Mediterranean; horizontal hatching–south-western LGM refugium; filled with colors–modern (mid-20th century). Note: Atlantic ocean, Mediterranean, Black and Azov sea max. regression, Caspian sea max. transgression coast line shown with the dashed line.

It is likely that these conditions coincided with the glacial period appropriate for the steppe faunistic complex expansion and might have led to the movement of the Common hamster from the North Mediterranean not only to the northwest, but also to eastern areas along the southern coast of the Black Sea to the Caucasian region. Indeed, late Pleistocene fossils of this species have been reported from territories located south of the main Caucasus Range on the Black Sea coast and in more eastern areas, such as Abkhazia, South Ossetia, and Imereti [53–56]. Fossils dated from the period at the end of the MIS-5 and the beginning of the MIS-4 epoch have been reported from southwestern France [57]. Thus, in the middle of the last glaciation period (Würm), the Common hamster’s range might have been continuous with the territory from modern southern France (where from hamsters might have extended their range to the northern region recently reported as the North phylogroup distribution area) to Caucasus (Fig 5).

This hypothesis, in our opinion, best explains the close relationship between the Caucasus and North-type lineages, whose ranges in the present day are separated by a great distance. Extinction of southern (North Mediterranean) populations presumably took place at the end of the LGM. In this area the most recent remains dated end of Late Pleistocene–early Holocene were found in Northern Italy [49, 58], Bosnia and Herzegovina, Slovenia, [50] and, possibly, in Northern Montenegro [59]. Remains of the Common hamster were found in Southeast Anatolia, Turkey were dated even from a later period– 5 kya, in the middle of the Holocene [60].

At the same time in South-East France the remains of the Common hamster are known both from Heinrich Stadial 1 (18–17 kya), and later in the Holocene [58]. It seems likely that more northern regions of Western Europe (Germany, Belgium, the Netherlands, i.e. the modern range of North phylogroup) were occupied by hamsters at the end of LGM from the South (France) but not from the East (Poland, Ukraine). Natural zone shifting from the South to the North caused by the Ice shield retreat caused the hamster range to drift northward and to vanish in southern and central France as well as from the North Mediterranean region.

The end of LGM (about 18 kya) was accompanied by the greatest Khvalynian Caspian transgression and New Euxinian transgression of the Black Sea [52]. The direct land corridor between the Mediterranean and Caucasus ceased to exist because of the Black Sea expansion and the Bosporus strait opened. At the same time, Caucasus was separated from the eastern East European Plain and Crimean Peninsula by the newly opened Manych Strait. These conditions forced the isolation of Caucasus (presumably originating from the Mediterranean) and eastern European (E0 phylogroup) hamster populations. However, independent evolution of the Caucasus and North-type lineages could have begun earlier because of the isolation by distance, despite continuous range.

The Common hamster belongs to the Late Pleistocene "Mammoth Fauna"–a complex of species distributed in the in periglacial open landscapes, which may have no analogous ecosystem in today's landscape. This complex includes large extinct species of mammals such as mammoth (*Mammuthus primigenius*), woolly rhinoceros (*Coelodonta antiquitatis*), etc., however, for this study we are mostly interested in small mammal species that have avoided extinction. During the Late Pleistocene–Early Holocene, these landscapes periodically expanded to include a considerable part of Europe all the way to Atlantic coast of France and the British Islands. In the Holocene, in the course of both climate warming and humidification, the open landscapes in Europe were replaced by forests. The ranges of a number of species that followed the retreating steppes shifted far to the East. However, some species (for instance, g. *Spermophilus*, g. *Sicista*, g. *Cricetus*, etc.) were retained in the relict steppe areas and other grasslands of southern and central Europe.

J. Stewart and co-authors (2010) [61] believe the Balkans, Turkey and Apennine peninsula to be glacial refugia for temperate species. However, the presence of open landscape species such as the grey hamster (*Cricetulus migratorius*) [62] and the European ground squirrel (*Spermophilus citellus*) in the Balkans, both in the Late Pleistocene and in the present day, allows scientists to consider these area also as an interglacial cryptic refugium for continentally adapted taxa. Persistence of a short-grass steppe-like refugium in the southern Balkans was also suggested by B. Kryštufek and co-authors (2009) [63]. The presence in southern France of not only the Common hamster but narrow-skulled vole (*Lasiopodomys gregalis*) and ground squirrel (*Spermophilus* sp.) until the Early Holocene [58] confirms that this region in LGM was also a refugium for the steppe-inhabiting complex of mammals.

It is noteworthy that the Common hamster is one of the most widespread species of the relic Mammoth Fauna in Central and Western Europe at present. We suggest that its success is associated with adaptations to large variety of habitats, not only steppe-like, but meadows, wastelands and shrubberies, and nowadays–agrocenosis. Indeed, *Cricetus cricetus* remains were often found together with broadly distributed and abundant species such as voles (*Microtus arvalis* and *M*. *agrestis*) and mouse (*Apodemus sylvaticus*) in Pleistocene deposits [48, 49, 57, 64].

It is more difficult to suggest the factors and paleoclimatic events that led to the isolation of ancestors of E0 and E1 phylogroups. Presumably, it also occurred after the Riss-Würm interglacial period (MIS-5 epoch) and, based on contemporary lineages distribution, the division took place in territories of Eastern Europe and Russia. Indeed, in the territory of modern Russia, not only Caucasus but also other refuge regions have been reported. Of these, the Ural region, Upper Dnieper River, and Central Russian Uplands of central Russia have retained high biodiversity [30]. Considering the patterns of modern distribution, the Ural region might have been the refuge where ancestors of E0 were isolated. This suggestion is confirmed by findings of *Cricetus cricetus* remains in Southern Urals almost throughout the entire late Pleistocene: at levels correlated with Riss-Würm interglacial period (Mikulino), MIS-3 epoch (Tabulda), and the beginning and end of the LGM [65, 66]. The grasslands of Russia and the Ukraine might have been an area where E1 arose, as it was presumed earlier [11, 25–28], but it requires an additional analysis.

There is no evident geographical and/or ecological barriers that might have isolated recent populations of E0 and E1 phylogroups from one another. The border between them apparently corresponds with the longitude of eastern Ukraine and the Sea of Azov (where the Crimean Peninsula is a part of the range of the E0 lineage). However, the phenomenon of sustained allopatry of different phylogenetic lineages of the same species or of closely related (able to crossbreeding under laboratory conditions) species occurring where proximate (sometimes separated by distance comparable with individual home ranges) locations exist is well known. One example is that of the allopatry of the Pannonia and E1 phylogroups of the Common hamster in Poland [26], and another one–allopatry of chromosomal races of striped hamster (*Cricetulus barabensis* sensu lato) [67].

The diversity and distribution patterns of phylogenetic lineages of the Common hamster within the territory of Russia and northern Kazakhstan is well correlated with the distribution of groups that differ by morphology and phenotypes. As stated above, the Ciscaucasius region is inhabited by *C*. *c*. *stavropolicus*. This form differs from *С*. *с*. *tauricus* that occurs in the territory between the Don and Dnieper rivers and in Crimea. The distribution of the E1-lineage hamsters covers some regions of Ukraine and Poland and it corresponds with the distribution of *С*. *с*. *nehringi* Matschie, 1901 described from this territory (to the west from right side of the Dnieper River). Finally, the wide distribution of the E0 lineage is in accordance with the distribution of *C*. *c*. *rufescens* [55]. This is in contradiction with a statement regarding the low diversity of the Common hamster known in eastern parts of the species range, as concluded by Kryštufek *et al*. [68]. The author statement "*The western segment of the Common hamster’s range (to the west of the Carpathian Mts*.*) is the most diverse genetically and morphologically while the populations to the east of the Carpathians are rather uniform*" appears to be incorrect.

The information regarding the current status of Common hamster populations that inhabit different regions and their genetic relatedness is important for protection and restoration of the Common hamster. The most dramatic population collapses have occurred along the western part of the species range. The Common hamster populations remain relatively stable in Romania [38], the territory of Russia, Ciscaucasia, and the Ural region. The origin of the Western European and Caucasian lineages from a common ancestor proposes Ciscaucasia as the donor region for the re-introduction of the species into western European countries.
